# Syphilinum

**Published:** 1891-01

**Authors:** Chas. B. Gilbert

**Affiliations:** Washington, D. C.


### SYPHILINUM.

Chas. B. Gilbert, M. D., Washington, D. C.

G. H., fifteen years old, has red hair, brown eyes, fair, somewhat freckled skin, and is of good height; was fat as a baby but has grown spare; in stature like his father, in complexion like his mother; the health of his father has always been poor, without any special evidence of disease; the second son died at the age of twenty-two (I think) of "consumption." As a child the patient had measles, whooping-cough, and mumps; in 1881 had ulcers on both shins and calves, which were healed with salves and which left depressed scars. In 1882 he had a low fever resembling typhoid. For some time he has been running down and now has bronchial breathing in the upper lobe of the right lung with prolonged expiration and crepitant râles; the heart's action is labored and the sounds, which are fairly distinct, are heard over an increased area on the right side; cough, but no expectoration; moaning in sleep; feet moist and at times hands also. Believing that the boy's taint was specific, he was given one dose of Swan's Syphilinum, very high, October 20th, 1889. On November 2d the lung sounds were better, there were no râles, no cough, and he looked better. He received no more or other medicine, and to-day (October 25th, 1890) his mother reports him still in good health.

This case, as well as many others, confirms Swan's generalization that a morbific product will cure a similar diseased condition in another person when given in a proper dose, which is simply the principle of vaccination. I have given Syphilinum repeatedly to the children of syphilitics and always with benefit. Its action seems to be similar to Psorinum (C. Hg.), which I believe to be nothing else than Syphilinum in a modified form, if it can be modified except in virulence.

I would say to that recent graduate on the back seat with his nose turned up, that the poison of man is no worse than the one he gave to that child the other day with such relief to its strangling whooping-cough—Mephitis.

